# Influences of pupal presence and worker number on the survival and development of pharaoh ant (*Monomorium pharaonis*) brood under laboratory conditions

**DOI:** 10.1080/19768354.2026.2657636

**Published:** 2026-04-18

**Authors:** Hee Jin Baek, Byunghyuk Kim

**Affiliations:** Department of Life Science, Dongguk University-Seoul, Goyang, Republic of Korea

**Keywords:** Brood rearing condition, Social composition, Colony demographics, Brood survival, Brood development

## Abstract

Social isolation and inadequate group composition can severely impair brood survival in eusocial insects due to the loss of social interactions. Establishing small artificial colonies is a prerequisite for studying developmental biology and functional genetics in ants; however, the minimal social requirements for effective brood rearing remain poorly understood. In this study, we investigated the impact of social composition – specifically the presence of pupae and worker abundance – on the survival and development of intact eggs within laboratory-maintained sub-colonies of the pharaoh ant, *Monomorium pharaonis*. Our analysis revealed that supplementing colonies with pupae significantly enhanced brood survival and accelerated larval development. Furthermore, we identified that sufficient worker numbers improve brood success, supporting the existence of a viable colony size. Our data suggest that while a sufficient workforce is essential for brood care, further increases in worker number do not yield proportional gains in rearing efficiency under our laboratory conditions. These results indicate that establishing favorable rearing conditions depends on achieving a functional demographic balance, particularly through pupal supplementation and an appropriate worker ratio. This study highlights the vital role of colony demographics and pupal presence in ant development, providing a practical framework for brood manipulation and ensuring baseline survival for future functional genetic applications.

## Introduction

Eusocial insects, particularly ants, are characterized by their advanced social organization and reproductive division of labor. A fundamental trait of ant biology is the extreme dependency of the brood on social care. Unlike precocial insects, ant larvae are altricial; they cannot feed or defecate independently and require constant grooming, feeding, and protection provided by nestmates to survive and develop (Hölldobler and Wilson 1990). These social interactions ensure that the colony maintains homeostasis, thereby protecting the brood from environmental fluctuations (Koto et al. 2015; Modlmeier et al. 2019). Consequently, the rearing environment – specifically the social composition of the colony – is a critical determinant of brood viability.

Unlike most ant species that establish new colonies with a solitary queen, highly polygynous invasive ants (e.g. Argentine ants and pharaoh ants) form new colonies by fragmenting a large source colony into smaller units that already contain a sufficient number of colony members and all essential social castes – queens, workers, and brood (Holway and Case 2001; Buczkowski and Bennett 2006; Buczkowski and Bennett 2009). This budding process is a highly effective local dispersal strategy that increases the likelihood of colony founding success, contributing to their invasiveness (Buczkowski and Bennett 2006). Notably, these budded colonies maintain stable caste ratios – eggs, larvae, pupae, workers, and queens – closely resembling those of the source colony and adhere to an optimal group size, underscoring the importance of adequate colony demographics for colony functionality (Buczkowski and Bennett 2009). Colony demographics and social structure critically influence overall colony fitness by enhancing social behaviors such as defense (Holway and Case 2001; Walters and Mackay 2005), foraging (Holway and Case 2001; Buczkowski and Bennett 2008), colony establishment (Hee et al. 2000), and the production of new workers and reproductives (Walin et al. 2001; Sorvari and Hakkarainen 2007). These characteristics of budding ant species suggest that optimized colony demographic factors, such as caste presence and colony size, ensure the minimal social interactions required to sustain colony function and brood development.

Understanding these minimal social requirements is not only ecologically significant but also methodologically critical for laboratory-based ant brood manipulation and functional genetic research. Recently, CRISPR-Cas9 genome editing has been applied to ants to study the genetic basis of ant physiology and social behavior (Trible et al. 2017; Yan et al. 2017; Chiu et al. 2020; Konu et al. 2023). In these protocols, treated ant embryos are reared to specific developmental stages in ‘small artificial colonies,’ which are intentionally fragmented from a large stock colony, especially for behavioral studies that require the brood to mature into fully functional adults (Trible et al. 2017; Yan et al. 2017; Konu et al. 2023). Introducing target brood into a stock colony is impractical because tracking the brood among numerous colony members is challenging. A major bottleneck in rearing brood within artificial colonies is the low survival rate of microinjected embryos, which are often fragile and prone to mortality. Distinguishing whether low survival is due to the toxicity of the editing reagents or suboptimal rearing conditions is difficult without a standardized rearing protocol.

Our model species, the pharaoh ant *Monomorium pharaonis*, is an invasive species native to tropical regions but now widespread indoors worldwide (Wetterer 2008). This species exhibits a monomorphic worker caste and is characterized by extreme polygyny (multiple queens), typically forming large colonies composed of 1,000–2,500 workers and 50–250 queens (Buczkowski and Bennett 2009; Schmidt et al. 2011), as well as a polydomous network maintained by contact among multiple nests (Oi et al. 2000; Buczkowski and Bennett 2009). Due to its status as an indoor nuisance pest, studying its ecological and physiological traits holds practical value. Pharaoh ants show several attributes advantageous for laboratory research. Notably, their extreme polygyny allows for the formation of high-density colonies within confined spaces (Buczkowski and Bennett 2009), which facilitates the efficient collection of large numbers of brood for experimental manipulation and the establishment of numerous small artificial colonies for brood management. Their unicoloniality permits the acceptance of non-nestmates without aggression, allowing flexible integration of colony members (Schmidt et al. 2010). In addition, queens and males mate within the nest (intranidal mating), simplifying the maintenance and propagation of colony lineages over generations (Schmidt et al. 2011). Beyond these ecological characteristics, key prerequisites for functional genetic studies, including a fully sequenced genome and a detailed developmental table, are already available for pharaoh ants (Gao et al. 2020; Rajakumar et al. 2024). Collectively, these features position the pharaoh ant as a promising new model system for genetic research. However, the specific social factors – such as a minimum number of workers or the presence of other brood stages like pupae – that are necessary to maximize brood survival in small laboratory sub-colonies remain to be determined.

In this study, we investigated the effects of social composition on brood survival and development in *M. pharaonis* using intact eggs. Our aim was to identify optimal conditions that promote efficient brood rearing in artificial colonies, with demographic factors serving as crucial determinants. Workers are known to play a central role in brood recognition and care in ant colonies (Schultner and Pulliainen 2020). Brood, especially pupae, also actively contribute to inclusive fitness and cooperative behavior important for colony development (Schultner et al. 2017; Snir et al. 2022). Here, we focused on two key variables: pupal supplementation and worker number. Other variables, such as larvae and queens, were excluded from our analysis to eliminate the risk of demographic overlap, as newly laid eggs or maturing larvae could not be reliably distinguished from our target cohort. We hypothesized that the presence of pupae and a sufficient workforce would provide essential nutritional and behavioral support, thereby enhancing brood survival through social interactions. We found that both pupal supplementation and sufficient worker numbers significantly improve brood growth efficiency.

## Materials and methods

### Stock colony maintenance

A colony of pharaoh ants (*M. pharaonis*), containing approximately 50 queens, was obtained from a private ant keeper in July 2023 (originally collected in Seoul, Republic of Korea). The colony was housed in a square plastic container (199 × 199 × 58 mm, 1.3 L) divided into four compartments by internal partitions. To form the nest floor, 30 mL of dental plaster (32 g powder mixed with 10 mL water) was poured to a depth of 5 mm and allowed to dry for one week. Each partition has a 1 cm diameter hole to allow free movement of ants between compartments. Fluon was applied to the upper inner walls to prevent escape. A piece of red transparent cellophane (7 × 5 cm) was folded to create a slight gap beneath it and placed inside the container to serve as a shelter, inducing ants to nest under it. The colony was maintained at 25°C under a 12:12 h light-dark cycle. Humidity was passively maintained by the water content in insect jelly (Cheongam & Insect Jelly); no additional water was provided. Approximately 1.5 g of insect jelly and five mealworms (*Tenebrio molitor*) were placed on 2 × 2 cm pieces of aluminum foil and provided as food every four days. The mealworms were cut in half and had their guts removed to prevent mold growth.

### Experimental colony maintenance

Experimental colonies were established in 60 × 15 mm Petri dishes (SPL Life Science). The inner walls were coated with Fluon, and the lids were covered with red cellophane. A folded piece of aluminum foil (3 × 4 cm) was used as a shelter to induce ants to nest underneath, as a cellophane piece was too light and easily displaced to provide a stable nesting site. All colonies were maintained at 25°C under a 12:12 h light-dark cycle. Humidity was provided by a 2.5 × 2.5 cm cotton pad soaked with 700 μL sterile distilled water, which was replaced every four days. Approximately 200 mg of insect jelly and one gut-removed mealworm were placed on 1 × 1 cm aluminum foil and supplied twice weekly.

For each experimental condition, nine replicates were established using sub-colonies sourced from the single stock colony. This approach was adopted to minimize genetic and environmental variance, thereby ensuring that observed developmental differences were primarily driven by the manipulated social factors. Experimental colonies were prepared by transferring workers from the stock colony, followed by a three-day stabilization period before each experimental test. Eggs, pupae, and workers were transferred using a damp brush. Queens were excluded from all experimental colonies to prevent mixing of the experimental eggs with those laid by queens.

### Pupal supplementation test

Queens were isolated in petri dishes to obtain eggs laid within 24 h. Of these, 10 eggs were introduced into each experimental colony, which contained 20 workers. For pupal supplementation, melanized pupae were transferred from the stock colony to the experimental colony. Pharaoh ant pupae are initially white but gradually turn yellow or reddish as they approach adult eclosion (Supplementary Figure 1A); these melanized pupae are reported to eclose within five days (Rajakumar et al. 2024). Therefore, any brood remaining in pre-adult stages (i.e. eggs, larvae, or pupae) after this period were considered to have originated from the introduced eggs. We provided three melanized pupae for pupal supplementation, as three pupae are expected for colonies with 20 workers based on the typical demographics of captive laboratory pharaoh ant colonies (Buczkowski and Bennett 2009; see Discussion). Pupal supplementation was repeated every five days and ceased once any brood reached the pupal stage (Supplementary Figure 1B). Throughout the experiments, the number of workers was adjusted every five days to maintain a constant total of 20 per colony (Supplementary Figure 1B). The number and developmental stage of surviving brood derived from the introduced eggs were recorded every five days. Dead brood were rarely observable, presumably due to desiccation or consumption by colony members, with the rare exception of dead pupae having rigid exoskeletons. We removed dead brood when occasionally found and only counted intact individuals as surviving brood. Data for reproductive – and worker-destined brood were separated from the second larval instar, when the caste fate could be visually distinguished based on morphological characteristics (Rajakumar et al. 2024). Due to experimental capacity constraints, nine colonies per condition (with or without pupal supplementation) were prepared across three separate sessions conducted in May – July, July – August, and October – December 2024. Kaplan-Meier survival analysis showed no significant differences in brood survival among the three sessions (log-rank test, *P* = 0.1793 for ‘with pupal supplementation’; *P* = 0.1770 for ‘without pupal supplementation’), indicating that seasonal variation was negligible.

### Worker number test

To assess the effect of worker number, 10 eggs laid within 24 h were introduced into each experimental colony, which contained either 5, 10, 20, or 40 workers. All colonies were supplemented with three melanized pupae, following the same protocol described above. Throughout the experiments, worker number was adjusted to maintain the initial worker number in each colony every five days. The number and developmental stage of surviving brood derived from the introduced eggs were recorded every five days. Data for reproductive – and worker-destined brood were separated from the second larval instar. For each group, nine colonies were prepared across three separate sessions. Kaplan-Meier survival analysis showed no significant differences in brood survival among the three sessions (log-rank test, *P* > 0.05 for all groups), indicating that seasonal variation was negligible.

### Estimating larval and pupal durations

Since physical marking of individual brood was not feasible in our experimental setup, we estimated developmental durations using a cohort-based approach. Throughout the pupal supplementation and worker number tests, we recorded the abundance and developmental stage of the surviving brood at each 5-day monitoring interval. From these census data, we estimated the stage duration as the midpoint between the minimum and maximum possible durations derived from the observation intervals. The average stage duration for each experimental colony was then calculated as the mean of these estimated durations. Our estimated larval and pupal durations in colonies without pupal supplementation (22.5 ± 5.0 and 11.9 ± 3.8 days, respectively; mean ± SD) align closely with previous developmental data for pharaoh ants under similar rearing conditions (approximately 22 and 12 days, respectively; Rajakumar et al. 2024).

### Statistical analysis

All statistical analyses were performed using GraphPad Prism 9. The Kaplan-Meier survival analysis was applied to compare survival rates across groups, and the differences were assessed using the log-rank (Mantel-Cox) test. The Mann-Whitney U test was used for comparisons between two groups. The Kruskal-Wallis test followed by Dunn’s post-hoc test was applied for comparisons among multiple groups.

## Results

### Pupal supplementation enhances brood survival

**Figure 1.**
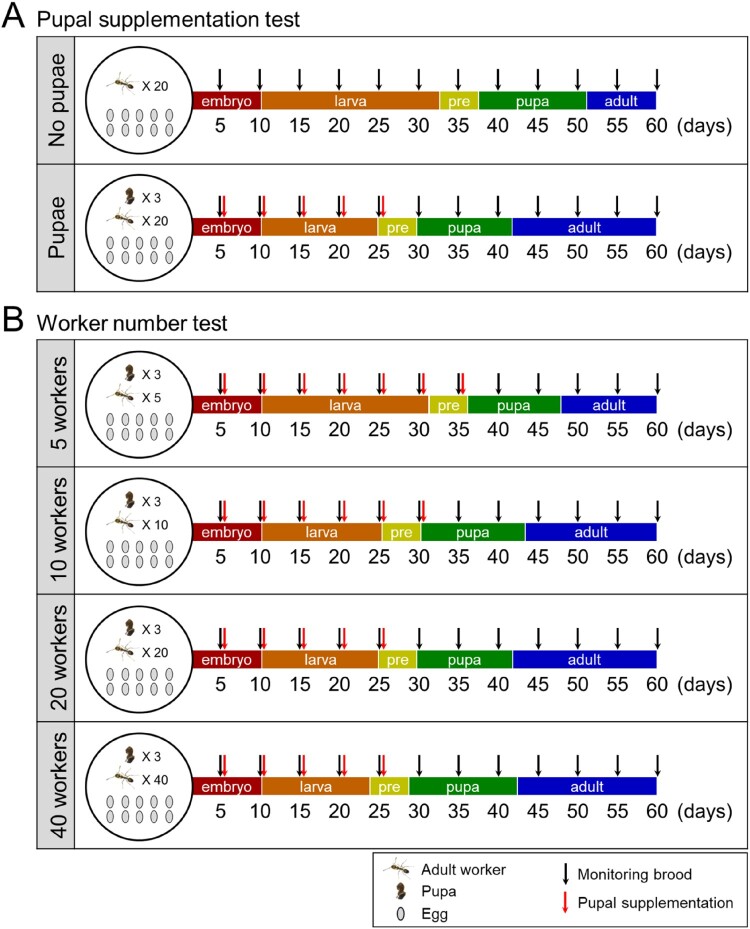
Experimental design and summary of brood development. Schematics of experimental setups comparing brood development in colonies with or without pupal supplementation (A) and with different worker numbers (5, 10, 20, or 40 per colony) (B). Nine colonies were prepared for each condition. Each colony was initially provided with 10 freshly laid eggs, the indicated number of workers, and three pupae, except for the ‘no pupae’ group. Fresh pupae were added every five days to maintain a constant number of three pupae per colony until any brood derived from the introduced eggs reached the pupal stage (red arrows). Initial worker numbers were kept constant throughout the experiments by adjusting worker numbers. Brood derived from the introduced eggs were counted, and their developmental stages were recorded every five days (black arrows). A summary of the developmental timeline was depicted based on the average duration of each developmental stage under each experimental condition. ‘pre’ indicates the prepupal stage.

**Figure 2.**
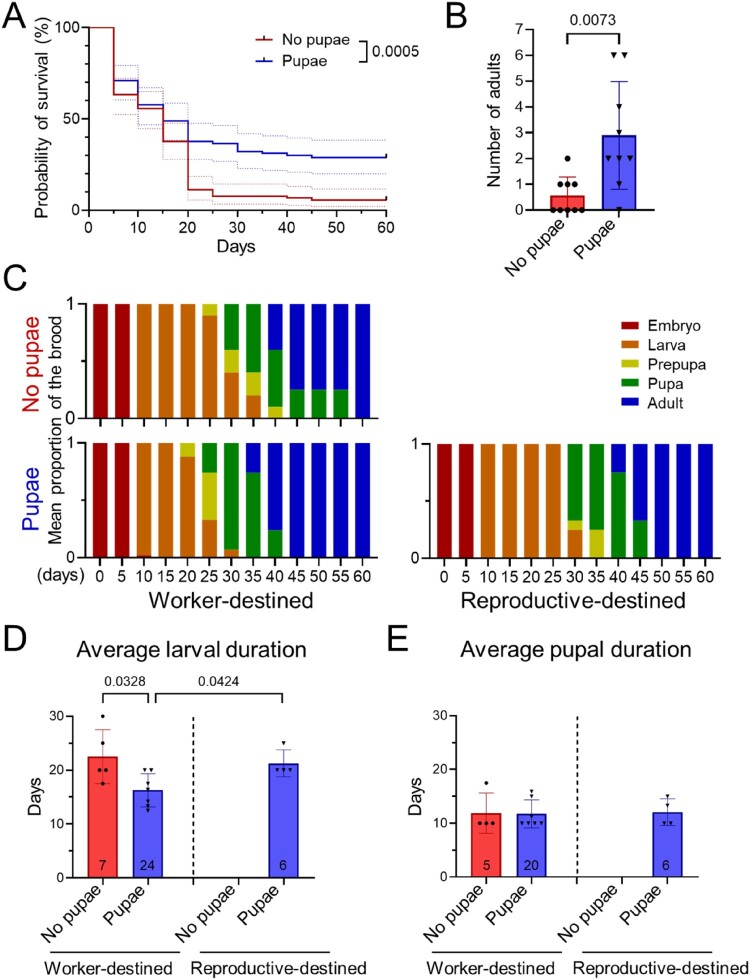
Effects of pupal supplementation on brood survival and development (A) Kaplan-Meier survival curves (solid lines) with 95% confidence intervals (dashed lines) for brood reared in colonies with or without pupal supplementation. Statistical significance was assessed using the log-rank (Mantel-Cox) test. (B) The number of adults successfully emerged from introduced eggs in colonies with or without pupal supplementation (mean SD). Mann-Whitney U test (n = 9 colonies per group). (C) Temporal changes in the mean proportion of brood in each developmental stage (colors) with or without pupal supplementation (n = 9 colonies per group). Data for worker- and reproductive-destined brood are presented separately due to differences in developmental rates. (D and E) Comparisons of average larval duration (D) and pupal duration (E) between colonies with and without pupal supplementation. Each dot represents the mean stage duration for a single colony (mean SD). The total number of individuals examined is indicated within each bar. *P*-values from the Mann-Whitney U test are shown in the graphs.

To test whether pupae influence brood survival and development, we set up experimental colonies containing 10 freshly laid eggs (within 24 h) and 20 workers, with or without three pupae (Figure 1A). Colonies with pupal supplementation showed higher brood survival rates compared to colonies without supplementation (Figure 2A). Survival analysis using the Kaplan-Meier method indicated a marked difference in survival rates between the two groups (log-rank test, *P* = 0.0005; Figure 2A), suggesting that brood survival is influenced by pupal supplementation. Similarly, the number of brood that successfully emerged as adults was significantly greater in supplemented colonies than in those without supplementation (Mann-Whitney U test, *P* = 0.0073; Figure 2B). Together, these results indicate that pupal supplementation substantially improves brood survival and their adult emergence.

### Pupal supplementation accelerates brood development

Brood in colonies with pupal supplementation also developed faster than those without supplementation. For each group, the average developmental stage distribution at each observation time point across nine experimental colonies was calculated and presented as the mean proportion of each stage (Figure 2C). The developmental stage distribution showed that transitions from larva to prepupa, prepupa to pupa, and pupa to adult occurred at least five days earlier in pupae-supplemented colonies (Figure 2C). The average duration of larval development was shorter in pupae-supplemented colonies (days ± SD: 16.3 ± 3.1 days) than in colonies without supplementation (22.5 ± 5.0 days; Mann-Whitney U test, *P* = 0.0328), whereas pupal duration did not differ between groups (approximately 12 days; Figure 2D and E). These results suggest that pupal supplementation primarily affects the larval stage, thereby speeding up overall brood development.

During the experiments, we occasionally observed some brood developing into reproductives (gynes or males) only in pupae-supplemented colonies. Due to the lack of distinct morphological traits to reliably differentiate between gynes and males during the larval stage (Rajakumar et al. 2024), we analyzed all reproductive-destined brood as a single group. While developmental durations may vary slightly between gynes and males, our primary objective was to contrast the developmental trajectories of reproductives against workers. The average larval period for reproductives (21.3± 2.5 days) was longer than for workers (16.3 ± 3.1 days), delaying their transition to the pupal and adult stages (Mann-Whitney U test, *P* = 0.0424; Figure 2C and D). However, there was no difference in the pupal period between worker – and reproductive-destined brood (approximately 12 days; Figure 2E). This result aligns with previous findings that reproductives generally require a longer developmental period than workers (Peacock and Baxter 1950). Whether pupal supplementation promotes reproductive production remains unclear and warrants further investigation.

### Worker number influences brood survival

**Figure 3.**
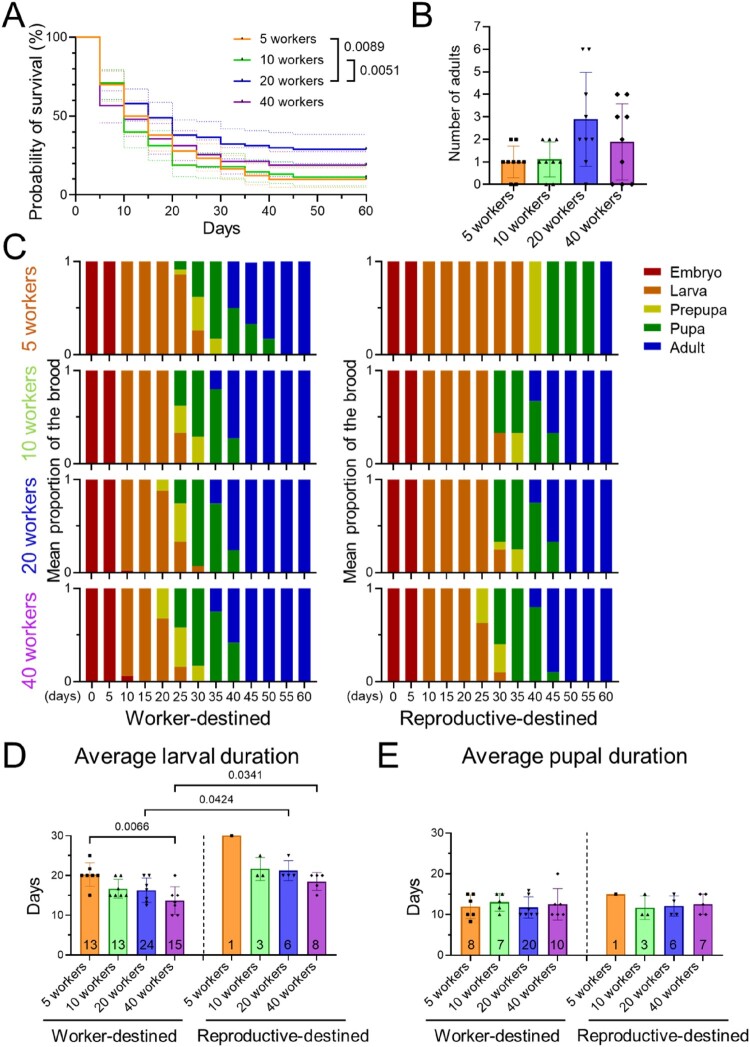
Effects of worker number on brood survival and development (A) Kaplan-Meier survival curves (solid lines) with 95% confidence intervals (dashed lines) for brood reared in colonies with 5, 10, 20, or 40 workers. Log-rank (Mantel-Cox) test. (B) The number of adults successfully emerged from introduced eggs according to worker number (mean SD; n = 9 colonies per group). (C) Temporal changes in the mean proportion of brood in each developmental stage (colors) in colonies with different worker numbers (n = 9 colonies per group). Worker- and reproductive-destined brood are shown separately. (D and E) Comparisons of average larval duration (D) and pupal duration (E) across colonies with different worker numbers. Each dot represents the mean stage duration for a single colony (mean SD). The total number of individuals examined is indicated within each bar. Kruskal-Wallis test followed by Dunn’s post-hoc test for comparisons among worker-destined groups; Mann-Whitney test for comparisons between the same worker-number groups. Only statistically significant *P*-values are shown in the graphs. The data for the 20-worker groups are identical to the pupae-supplemented groups presented in Figure 2.

To examine the effect of worker number on brood survival and development, we established experimental colonies with 10 freshly laid eggs and 5, 10, 20, or 40 workers (Figure 1B). Survival analysis revealed a significant difference in brood survival rates among the four groups (log-rank test, *P* = 0.0189; Figure3A). Colonies with 20 workers maintained higher brood survival rates over colonies with 5 workers (log-rank test, *P* = 0.0089) and 10 workers (log-rank test, *P* = 0.0051; Figure 3A). While the number of brood that successfully emerged as adults exhibited an increasing trend in colonies with 20 workers, the difference among groups did not reach statistical significance under the Kruskal-Wallis test (*P* = 0.0991; Figure 3B), which accounts for variance among replicates. However, when data were pooled across replicates within each group to assess overall eclosion success, pairwise comparisons using Fisher’s exact test revealed that the success rate was significantly higher in the 20-worker group than in either the 5-worker group (*P* = 0.0023) or the 10-worker group (*P* = 0.0047). These results suggest that worker number influences overall brood survival and the likelihood of reaching adulthood.

### Worker number modulates brood developmental speed

We next examined the distribution of developmental stages across colonies with different worker numbers. The mean proportion of developmental stages showed that worker-destined brood from colonies with fewer workers exhibited delayed stage transitions, with a similar pattern for reproductive-destined brood (Figure 3C). This difference in developmental speed appeared to be reflected in different larval development durations. For worker-destined brood, colonies with 5 workers had significantly longer larval periods (20.2 ± 3.0 days) compared to those with 40 workers (13.7 ± 3.5 days; Kruskal-Wallis test with Dunn’s post-hoc, *P* = 0.0066), while other pairwise comparisons did not reach statistical significance (Figure 3D). For reproductive-destined brood, we observed a similar trend but with extended larval durations. Pairwise comparisons between corresponding worker-number groups showed longer larval durations in reproductive-destined brood; however, statistically significant differences were only observed in the 20- and 40-worker groups, likely due to the small sample sizes in groups with fewer workers (Figure 3D). Pupal duration, however, did not differ significantly among groups for either caste (approximately 12 days; Figure 3E). These results suggest that worker number primarily affects brood development during the larval stage, consistent with the effect observed for pupal supplementation.

## Discussion

Our results demonstrate that social composition significantly modulates brood rearing efficiency in *M. pharaonis*. We identified that a specific combination of social factors – pupal supplementation and a worker population of 20 individuals – creates an efficient environment for experimental use, achieving sufficient embryo survival and adult emergence. These findings highlight the importance of social interactions in laboratory rearing systems and establish a foundational baseline for brood manipulation and functional genetic experiments.

### The role of pupae in brood development

We found that pupal supplementation dramatically increased brood survival and accelerated development (Figure 2). These effects are likely due to social interactions between brood and supplemented pupae, mediated by workers. In some eusocial insects, the presence of brood has been shown to influence colony members by regulating worker behavior and gene expression (Ravary et al. 2006; Eyer et al. 2017; Chandra et al. 2018). Such behavioral modifications can improve overall colony fitness and efficacy, for instance, by enhancing nursing capacity. If this is the case, our pupal supplementation is expected to yield the same effect. Technically, employing pupae specifically for brood supplementation offers an advantage in qualitative control, as pupae exhibit less size variation than larvae, which vary widely in size and instar stage. Moreover, using melanized pupae near eclosion for supplementation aids in the identification of brood derived from introduced eggs (see Materials and Methods).

A recent study has shown that ant pupal molting fluid plays a key role in colony interactions and contains nutritious organic compounds that enhance larval growth and survival (Snir et al. 2022). Molting fluids secreted by pupae are mainly consumed by young larvae, increasing the body size and survival rate of the larvae (Snir et al. 2022). This finding aligns with our observation that pupal supplementation primarily benefits the larval stages (Figure 2). In our experiments, the presence of pupae likely provided a supplemental nutritional source (i.e. social fluids) that workers delivered to the developing larvae, thereby reducing developmental time and mortality. This suggests that nutritional networks in *M. pharaonis* involve complex interactions among brood in various stages, as seen in other ant species (Schultner et al. 2017). Although we have not yet directly observed pupal secretions in *M*. *pharaonis*, future work should clarify the role of pupae and their secretions in promoting brood development in this species.

### Viable colony size, demographics, and worker number

Our study also reinforces the critical role of workers in the brood development of social insects. Worker-mediated brood care strongly influences the growth of ant brood as widely documented in eusocial insects (Linksvayer 2007; Chouvenc and Su 2017; Psalti et al. 2021). Beyond the mere presence of workers, having a sufficient worker number is crucial for efficient division of labor, which accelerates brood development (Ulrich et al. 2018; Bell-Roberts et al. 2024). This aligns with our observations: colonies with 20 workers exhibited survival metrics more than double those of colonies with 10 workers, and larval duration decreased as worker number increased (Figure 3). Consistent with our pupal supplementation results, worker number primarily influenced larval but not pupal durations. This was likely due to enhanced nutritional provisioning to larvae – the brood stage most sensitive to feeding efficiency (Nestel et al. 2016) – through the improved task performance of workers. In budding species like *M. pharaonis*, small groups must possess a critical mass of workers to perform essential tasks such as foraging, brood care, and nest maintenance effectively (Buczkowski and Bennett 2009). The poor performance of colonies with fewer workers, particularly the 5-worker group, suggests that the labor demand per worker may exceed their capacity, leading to neglected brood care. Interestingly, increasing the number to 40 workers yielded no further improvements in the survival or adult emergence of brood, suggesting that the demographic balance within the colony may be more critical than a mere increase in worker number.

Maintenance of artificial colonies with ideal demographics – such as containing all caste components or adjusting worker numbers proportional to the introduced brood – would be advisable (Warner et al. 2018). However, practical constraints, including technical limitations related to time and labor for artificial colony establishment and experimental control, must also be considered. Our preliminary tests, which varied worker numbers without pupal supplementation and strict control of introduced egg numbers, suggest that colonies with approximately 20 workers raised brood most effectively, possibly representing a favorable colony size in our experimental conditions. The number of supplemented pupae (n = 3) corresponds proportionally to 20 workers according to standard caste ratios of pharaoh ant colonies (eggs 12.8%, larvae 5.3%, pupae 10.0%, workers 66.6%, and queens 5.2%; Buczkowski and Bennett 2009). Thus, colonies with 20 workers satisfied stable demographics at least for the pupae:workers ratio, potentially explaining their superior brood survival. Over time, the reproductives produced in some 20-worker groups might further shift colony demographics toward standard caste ratios (Warner et al. 2018). Experiments adjusting the number of supplemented pupae to standard caste ratios across varying worker numbers could help test this hypothesis. Colonies constructed with demographics based on egg numbers (e.g. 52 workers and 7.8 pupae for 10 eggs) would also be useful for further investigation. As a baseline to determine an ideal sub-colony composition for rearing conditions, we recommend that researchers measure caste ratios in their own stock colonies or refer to published demographic data for the species.

These findings regarding worker number and the strong influence of demographic factors on artificial colony performance may stem from pharaoh ants’ ecological traits shaped by their budding-based nest-multiplication strategy. Unlike many eusocial species that initiate colonies from a solitary queen or few workers – thus experiencing imbalanced demographics during founding – pharaoh ants establish new colonies with sufficient numbers of all castes by budding (Buczkowski and Bennett 2009). This natural history may render pharaoh ants particularly sensitive to demographic imbalances in artificial colonies, resulting in performance differences based on demographic completeness. This hypothesis could be tested by conducting similar experiments with non-budding ant species, which might exhibit reduced sensitivity to variations in supplementary pupae or worker numbers. Overall, our findings emphasize the significant influence of pupae and worker numbers on brood growth efficiency and highlight the need for careful consideration of demographic factors in rearing brood in artificial colonies.

We propose that stable colony demographics improve colony functionality by modulating collective social behaviors and enhancing the efficacy of social interactions among colony members, as indicated by prior studies (Hee et al. 2000; Holway and Case 2001; Walin et al. 2001; Walters and Mackay 2005; Sorvari and Hakkarainen 2007; Buczkowski and Bennett 2008). Behavioral analyses – such as quantifying behavioral frequencies and tracking individual trajectories – under varied colony conditions could clarify the relationship between social interactions and colony demographics, particularly pupal presence and worker number (Modlmeier et al. 2019). This approach will help understand how colony composition influences social dynamics and overall colony performance.

### Production of reproductive individuals in a laboratory rearing system

Interestingly, both pupal supplementation and increased worker numbers positively influenced the production of reproductives. No reproductives emerged in colonies without pupal supplementation, while colonies with pupae and higher worker numbers produced reproductives more frequently (Figures 2 and 3). In *M*. *pharaonis*, the presence of fertile queens appears to suppress the production of new reproductives via queen pheromones (Edwards 1987; Edwards 1991; Oliveira et al. 2020). The earliest molecularly detectable time point for caste determination in this species is suggested to be Stage 5 embryos, which correspond to 25-36 h after egg laying (Rajakumar et al. 2024). Thus, the absence of queens in our experimental setup may have allowed the emergence of new reproductives, but their successful maturation likely required suitable rearing conditions. Previous studies have shown that caste differentiation in ants is influenced by multiple factors, including nutrition, temperature, and colony size (Smith et al. 2008; Trible and Kronauer 2017; Negroni and LeBoeuf 2023). It is plausible that stabilization of colony demographics by pupal supplementation and increased worker numbers enhances the efficacy of social interaction and subsequently improves brood nutritional status, thereby promoting reproductive production. Beyond elucidating the mechanisms inducing reproductive castes, our findings show that these rearing conditions in artificial colonies enable reproducible production of reproductives. This could be valuable for establishing stable genetic lines in genetic research. Further work to identify additional conditions that favor reproductive production will help advance functional genetic studies in ants.

### Implications for future genome editing research

While this study utilized intact, uninjected eggs, the implications for genome editing are also substantial. Previous CRISPR-Cas9 genome editing studies in ants have reported low hatching and eclosion rates, often attributed to injection trauma (Chiu et al. 2020; Konu et al. 2023). However, our data suggest that suboptimal social context may also be a major contributing factor to brood mortality. By applying the improved rearing conditions identified here, researchers can minimize background mortality caused by environmental stress. This ensures that any observed lethality in future experiments can be more accurately attributed to the gene editing process itself rather than poor rearing conditions. Thus, this protocol serves as a practical prerequisite step for improving the efficiency and reproducibility of molecular genetics research in ants.
